# Scaling of a biophysical neocortical attractor model using Parallel NEURON on the Blue Gene /P

**DOI:** 10.1186/1471-2202-12-S1-P191

**Published:** 2011-07-18

**Authors:** David Silverstein, Anders Lansner

**Affiliations:** 1 Department of Computational Biology, Royal Institute of Technology, Stockholm, Sweden; 2 Stockholm Brain Institute, Stockholm, Sweden

## 

This work entails scaling a biophysical model of the neocortex using parallel NEURON [1] while running on a Blue Gene / P in virtual node mode. Previous scaling experiments have been done with the SPLIT simulator on the Blue Gene / L with a similar neocortical model [2]. We chose a biophysical model of medium complexity based on the Hodgkin-Huxley formalism because this provides the capability of exploring the effects of psychotropic drugs as well as the oscillatory effects of cortical microcircuits and globally correlated network activity. Neocortical simulations were performed to determine both strong (fixed network size, increasing cores) and weak (increasing network size, fixed load per core) scaling with two variations of a square necortical patch of hypercolumns and internal minicolumns. The first variation consists of minicolumns with 20 layer 2/3 pyramidal cells, 2 basket cells and 5 layer 4 pyramidal cells and has orthogonally stored memory patterns, encoded with long-range excitatory connections between individual minicolumns across hypercolumns. The second variation has an additional 2 regular spiking non-pyramidal interneurons per minicolumn and instead uses sparse, randomly overlapping memory patterns encoded with both excitatory and inhibitory long-range connections between hypercolumns. Simulations were performed with both single patches of increasing area and cascades of multiple patches with feed-forward and feed-backward projections.

Individual simulations consisted of stimulation and completion of a single memory pattern within 1 second of cortical activity. Preliminary results show near linear speedups of the computational part of the simulation, but degradation of file I/O performance as the number of cores increase. Since each core writes out spiking activity after the simulation, the performance decline may be due to the ratio of core to I/O nodes and the large number of output files. With this performance analysis, further work will include measuring and scaling memory storage capacity with the described second variation of the biophysical neocortical model.

**Figure 1:**
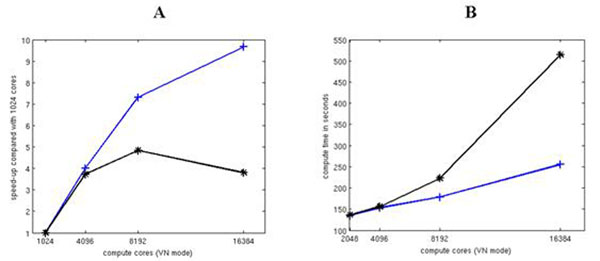
Strong and weak scaling results from simulations of the first variation of the neocortical model. The blue lines represent combined initialization and simulation time and the black lines also include writing spiking output. **A.** Strong scaling of a 16x16 hypercolumn neocortical patch with 128 minicolumns. **B.** Weak scaling of 4x4, 8x8, 4x8 and 16x16 hypercolumn patches all with 128 minicolumns. Each core computed 2 minicolumns.
